# Urinary Proteomics as a Noninvasive Readout of Obesity-associated Inflammation

**DOI:** 10.1210/jendso/bvaf212

**Published:** 2025-12-23

**Authors:** Esra Canki, Esther Kho, Ruud van Stiphout, Niels P Riksen, Mihai Netea, Leo A B Joosten, Rinke Stienstra, Joost Hoenderop

**Affiliations:** Department of Medical BioSciences, Radboud Institute of Medical Innovation, Radboudumc, 6525 GA Nijmegen, The Netherlands; Imec within OnePlanet Research Center, 6708 WE Wageningen, The Netherlands; Imec within OnePlanet Research Center, 6708 WE Wageningen, The Netherlands; Department of Internal Medicine, Radboudumc, 6525 GA Nijmegen, The Netherlands; Department of Internal Medicine, Radboudumc, 6525 GA Nijmegen, The Netherlands; Department of Immunology and Metabolism, Life and Medical Sciences Institute, University of Bonn, 53113 Bonn, Germany; Department of Internal Medicine, Radboudumc, 6525 GA Nijmegen, The Netherlands; Department of Medical Genetics, Iuliu Hatieganu University of Medicine and Pharmacy, 400347 Cluj-Napoca, Romania; Department of Internal Medicine, Radboudumc, 6525 GA Nijmegen, The Netherlands; Division of Human Nutrition and Health, Wageningen University, 6708 PB Wageningen, The Netherlands; Department of Medical BioSciences, Radboud Institute of Medical Innovation, Radboudumc, 6525 GA Nijmegen, The Netherlands

**Keywords:** OLINK, targeted proteomics, obesity, BMI, urine, biomarkers

## Abstract

**Introduction:**

Obesity affects a large percentage of the population, and it is associated with many comorbidities. These pathologies often coincide with the presence of a low-grade chronic inflammatory state in individuals with obesity that can be measured using circulating inflammatory proteins. The objective of this study was to use a noninvasive approach to determine obesity-induced inflammation by measuring the urinary proteome of individuals with and without obesity.

**Methods:**

Morning urine samples were taken from normal-weight controls (n = 30) with a mean body mass index (BMI) of 22.8 kg/m^2^ and people with obesity (n = 58) with a mean BMI of 32.9 kg/m^2^. Further data regarding high-density lipoprotein, fat distribution, blood pressure, and waist circumference were available from the individuals with obesity. Using these samples, normalized protein expression data were obtained using the Olink Explore 384 inflammation panel. These data were analyzed using R to elucidate the differences in protein expression between the individuals with and without obesity.

**Results:**

Of the 384 inflammation proteins, 48 proteins had a *P* < .05 with false discovery rate correction between the persons with obesity and the persons without obesity. Network analysis revealed 5 different clusters of proteins with several clusters associated (*P* < .05) with circulating concentrations of high-density lipoprotein, waist circumference, and fat distribution in individuals with obesity.

**Conclusion:**

This paper shows that urine may represent a novel noninvasive approach to measure the state of inflammation in individuals with obesity using Olink targeted proteomics.

Obesity and overweight are growing public health concerns worldwide, affecting 60% of adults and almost 1 in 3 children in Europe [1], and are major contributors to several diseases [2]. Obesity can cause metabolic syndrome, which leads to different symptoms including increased blood pressure, dyslipidemia, raised fasting glucose, and an increased waist circumference. Metabolic syndrome greatly increases the risk for the development of cardiovascular disease and type 2 diabetes mellitus in individuals with obesity [3-5]. The presence of obesity is often accompanied by a low-grade inflammatory state that has been shown to largely to originate from expanding adipose tissue. Extensive research has already shown that macrophages and the adipose tissue themselves are 1 of the main causes of proinflammatory cytokine release [6, 7]. However, other components of the immune system are also affected, as the number of white blood cells is increased in people with obesity and concentrations of C-reactive protein (CRP) produced by the liver are increased [8-10]. The neutrophils and monocytes are present in the adipose tissue and perpetuate the inflammatory state in the body by releasing cytokines and chemokines [11]. Different organs are involved in the low-grade inflammatory state in the body. The liver produces cytokines and chemokines, while the intestines become more permeable [12, 13]. This leads to an increase in circulating microbial products, such as lipopolysaccharide and muramyldipeptide from bacteria in the intestines, causing an immune response and increasing the concentrations of cytokines and chemokines [12]. Thus, many organ systems are involved and play a role in maintaining the low-grade inflammatory state in the body.

Often, biomarkers in the blood or adipose tissue are used to determine the state of chronic inflammation during obesity, which requires blood sampling or adipose tissue biopsies. Common biomarkers in serum include cytokines released by the adipose tissue, including IL-6, TNFα, and CRP [14]. However, these methods are invasive and difficult and often have many risks attached to them. In spite of this, recognizing chronic inflammation in obesity is crucial in the monitoring and treatment of complications associated with obesity [15]. There is limited published data on establishing the presence of chronic inflammation during obesity using a noninvasive manner. A previous study investigated the urine of metabolically unhealthy participants using mass spectrometry [16]. Urinary testing could provide a noninvasive way of determining and monitoring the chronic inflammation of individuals with obesity; thus it is necessary to further investigate urinary proteins. As the collection is noninvasive, urine can provide a method by which the individual can easily collect the sample themselves. Therefore, we set out to explore changes in urinary inflammation proteins by comparing urine samples from normal-weight controls and individuals with obesity.

## Material and Methods

### Study Population

As the study is exploratory in nature, a smaller sample size was used to determine whether differences would be observed. Urine samples of 88 individuals were used, including n = 58 samples of individuals with obesity [body mass index (BMI) > 30] and n = 30 samples of study participants without obesity (BMI 20-25) matched on sex and age (Table S1) [17]. The samples were obtained from 2 existing cohorts. The samples of individuals with obesity came from the 300OB cohort [18, 19]. The samples of the control group, individuals without obesity, came from the 500FG cohort [20]. Both cohorts were described previously (www.humanfunctionalgenomics.org) [17]. Tables 1 and 2 describe the characteristics of samples used in this study from the 300OB cohort and 500FG cohort, including age, sex, BMI, glucose levels, and fat distribution [17]. Both studies were approved by the Ethical Committee of the Radboudumc (300OB, NL46846.091.13 and 500FG, NL42561.091.12, 2012/550) and conducted in accordance with the principles expressed in the Declaration of Helsinki. In short, inclusion criteria for the 300OB cohort included age older than 55 years and a BMI greater than 27 kg/m^2^. All women in the 300OB cohort were postmenopausal and were not receiving hormonal replacement therapy. Exclusion criteria for this cohort included recent cardiovascular events, a history of bariatric surgery, inflammatory bowel disease, and renal dysfunction. The 500FG cohort consists of healthy participants of Caucasian origin. Participants were included if they did not have an acute or chronic condition at the time of participation and did not use medicine. They were excluded if their age was < 18 years or they were pregnant.

**Table 1.** Characteristics of the 300OB cohort, including age, BMI, waist circumference, and fat distribution

	Female n = 30*^a^*	Male n = 30*^a^*	*P*-value*^b^*
Age (years)	63.5 (3.2)	61.6 (3.3)	.035
BMI (kg/m^2^)	33.4 (3.6)	32.5 (2.1)	.3
Waist circumference (cm)	108.1 (9.4)	114.4 (7.1)	.006
Waist-hip-ratio (cm)	0.9 (0.1)	1.0 (0.1)	<.001
Systolic blood pressure (mmHg)	131.1 (15.4)	132.5 (13.3)	.7
Diastolic blood pressure (mmHg)	78.6 (9.0)	85.3 (9.1)	.008
Presence of plaques (%)			>.9
No	15.0 (50.0)	14.0 (50.0)	
Yes	15.0 (50.0)	14.0 (50.0)	
Fat distribution (VAT/VAT + SAT)	0.3 (0.1)	0.4 (0.1)	<.001
HbA1c (mmol/mol)	43.8 (9.7)	39.6 (4.7)	.041
Presence of hypertension (%)			.060
No	6.0 (20.0)	12.0 (42.9)	
Yes	24.0 (80.0)	16.0 (57.1)	
Presence of diabetes mellitus (%)			.7
No	25.0 (83.3)	25.0 (89.3)	
Yes	5.0 (16.7)	3.0 (10.7)	
Antihypertensive medicine use (%)			.034
No	12.0 (40.0)	19.0 (67.9)	
Yes	18.0 (60.0)	9.0 (32.1)	
Antidiabetic medicine use (%)			>.9
No	27.0 (90.0)	26.0 (92.9)	
Yes	3.0 (10.0)	2.0 (7.1)	
Lipid-lowering medicine use (%)			.3
No	27.0 (90.0)	22.0 (78.6)	
Yes	3.0 (10.0)	6.0 (21.4)	
MetS score (%)			.3
1	1.0 (3.3)	2.0 (7.1)	
2	10.0 (33.3)	9.0 (32.1)	
3	5.0 (16.7)	10.0 (35.7)	
4	9.0 (30.0)	3.0 (10.7)	
5	5.0 (16.7)	4.0 (14.3)	

Abbreviations: BMI, body mass index; HbA1c, hemoglobin A1c; MetS, metabolic syndrome score; VAT/VAT+SAT, visceral adipose tissue/visceral adipose tissue + subcutaneous adipose tissue.

^*a*^Mean (SD); n (%).

^*b*^Welch 2-sample *t*-test; Pearson's chi-squared test; Fisher's exact test.

**Table 2.** Characteristics of the 500FG cohort, including the age and BMI of the participants

	Female n = 15*^a^*	Male n = 15*^a^*	*P*-value*^b^*
BMI (kg/m^2^)	22.4 (1.7)	23.2 (1.4)	.2
Age (years	60.4 (5.4)	64.5 (3.5)	.022

Abbreviations: BMI, body mass index.

^*a*^Mean (SD).

^*b*^Welch 2-sample *t*-test.

#### Sample selection

Firstly, in the 300OB selection, those with BMI ≤ 30 were excluded. Second, 29 with and 29 without plaques were selected, and these groups were age- and sex-matched. Finally, from the healthy controls (500FG) cohort, 28 participants were selected, with a BMI between 20 and 25, which were age- and sex-matched with the other 300OB groups.

#### Sample collection

In both cohorts, urine and blood samples were collected in the morning. Aliquots of urine were stored at −80 ^o^C for both studies, and a fresh aliquot was thawed and used for the current study. Blood samples in the 300OB cohort were collected after an overnight fast. The blood samples were used to determine fasting blood glucose, triglycerides, and high-density lipoprotein cholesterol according to standard procedures. Furthermore, systolic and diastolic blood pressure were measured after rest. Subcutaneous adipose tissue was obtained under anesthesia, and the morphology of the fat cells was determined using digital image analysis as described previously [19]. A subselection of the cohorts was made based on age and sex to age- and sex-match the cohorts.

### Olink Analysis

Urine samples were analyzed using the Olink Explore 384-inflammation panel, consisting of 384 inflammatory proteins. In short, 40 μL urine was diluted 10× and samples were randomized before measurements were done (Fig. S1) [17]. Per biomarker in the inflammation panel, Olink provides a relative concentration expressed in the normalized protein expression value (NPX). To account for dilution differences between the urine samples, NPX values were normalized using the creatinine concentrations from the urine that were measured by the Laboratory Medicine Department (Radboudumc). Normalization of the protein concentrations was achieved by subtracting the protein concentration from the creatinine concentration, as both were in log2.

### Statistical Analysis

All statistical analyses were performed using R Statistical Software version 4.4.2 [21]. Data was preprocessed, and samples below the limit of detection and samples with a quality control warning were removed. Due to the exploratory nature of this study, analyses were done without controlling for any additional confounders. First, a principal component analysis was conducted to identify and visualize differences in the measured biomarker profiles between the 2 groups (individuals with obesity n = 58 and individuals without obesity n = 28), allowing for the reduction of dimensionality and highlighting key patterns in the data. Second, these differences were further analyzed using individual *t*-tests, of which the output was corrected for multiple testing with the false-discovery rate (FDR), given the large number of tests (n = 384 proteins). These results were further interpretated using a volcano plot, a representation that allows for filtering proteins based on fold change (FC) and significance. The Log2FC was calculated by subtracting the control NPX values with the NPX of the individuals with obesity, and proteins were colored based on significance (*P* < .05 FDR-corrected and Log2FC <−1 or >1). Third, both subjects and proteins were clustered using hierarchical clustering based on protein expression, and results were interpreted in a heatmap. Fourth, the R package WGCNA [22] was used to determine the protein-protein interactions in the whole dataset. A scale-free topology of 0.88 was reached using a soft power of 2. The minimal cluster size was set to 5, as the dataset does not contain a large number of proteins. Clustering was achieved by creating a coexpression network using adjacency and TOMsimilarity of the WGCNA package. The clusters were then visualized using Cytoscape [23]. The clusters that were found using WGCNA were then used to determine the correlations between the clusters and the different risk factors for metabolic syndrome in the cohort of the individuals with obesity (n = 58) (waist circumference, systolic and diastolic blood pressure, fasting glucose, triglycerides, and high-density lipoprotein). Last, to investigate the significant (*P* < .05) proteins from the volcano plot and to determine the biological pathways that these proteins are involved in, an overrepresentation analysis (ORA) was used. This is a statistical method that determines whether a specific biological pathway is overrepresented in the enriched proteins. This is achieved by determining the biological pathway each gene is involved in, and the biological pathways with the most expressed genes are visualized. For the ORA, the R package clusterProfiler [24] with a Benjamini-Hochberg post hoc test was utilized.

## Results

**Figure 1.**
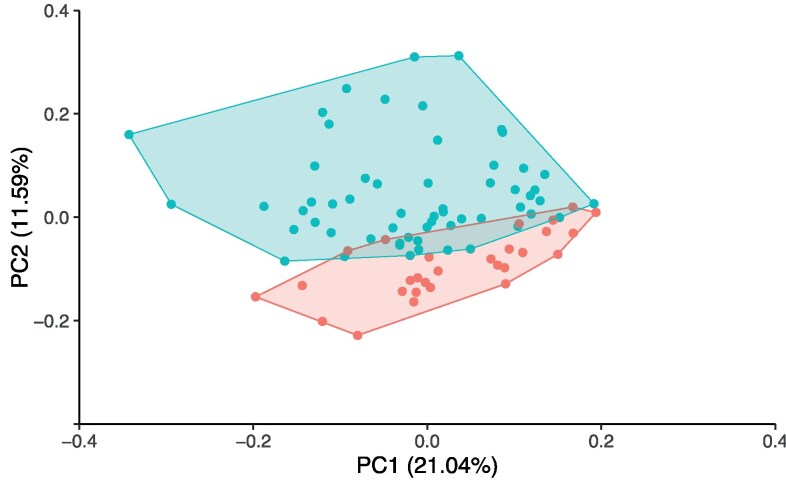
PCA shows the separability of the 2 groups. The PCs show the variance (32.69 of the variance) of the people with obesity (blue; n = 58; Table 1 for more information on this group) and the normal-weight controls (red; n = 30; Table 2 for more information about this group). PCs are expressed in the percentage variance. Dots represent the individual samples (n = 88). Abbreviations: PC, principal component; PCA, principal component analysis.

The first 2 components of the principal component analysis on the expression of 384 proteins are visualized in Fig. 1. This plot shows similarities between groups of samples in a data set using the 384 measured inflammation proteins. Thirteen of the individuals with obesity and 4 of the healthy-weight individuals were misclassified based on overlap between the clusters. The separation between the groups was mostly based on PC2, with the highest loadings for PC2 being ALDH3A1, BSG, and BTN2A1, among others (Fig. S2) [17].

**Figure 2.**
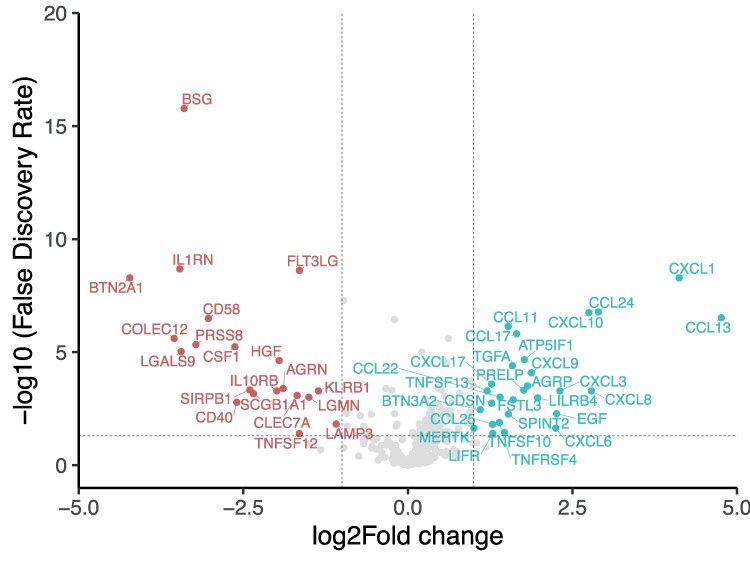
A volcano plot indicating the difference in urine protein levels in individuals with obesity compared to those without obesity. Significantly different (*P* < .05) proteins are either enriched in individuals without obesity (red n = 20; *P* < .05 and fold-change < −1), and individuals with obesity (blue n = 58; *P* < .05 and fold-change > 1) or are not significant (grey).

**Figure 3.**
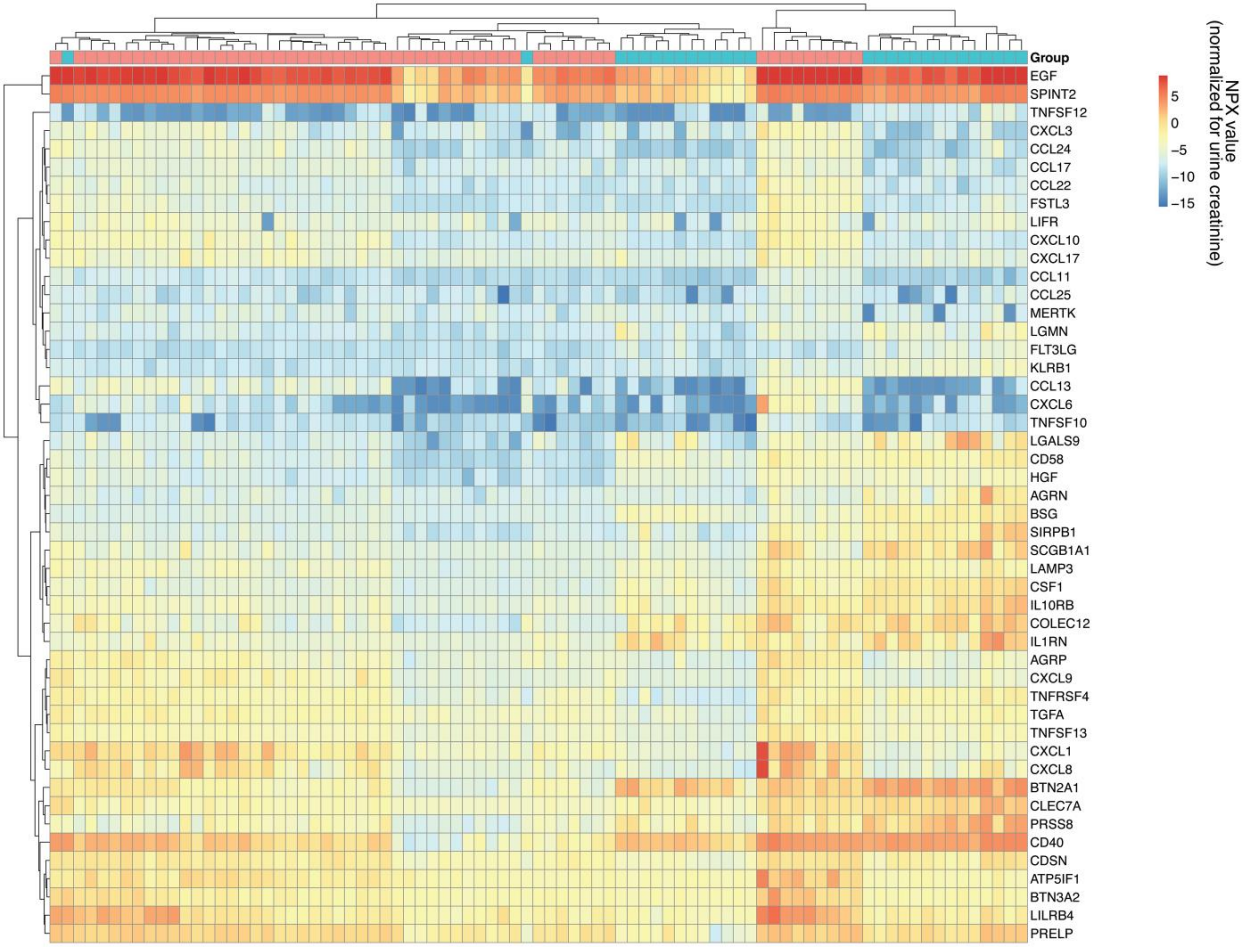
Heatmap analysis of the enriched proteins shows specific clustering in protein expression levels between the individuals without obesity (top bar; red n = 20) and individuals with obesity (top bar; blue n = 58). Red indicates an upregulation of certain proteins, while blue indicates a downregulation based on the normalized protein expression value. The dendrogram on the side and top shows the correlation between the different groups (top) and the proteins (left side).

Of the 384 proteins in the Olink inflammation panel, 48 were significantly different between the control group and the overweight group. The effect size and significance from the FDR-corrected *t*-tests are shown in a volcano plot in Fig. 2 and their expression in a heatmap in Fig. 3. The heatmap shows almost perfect clustering between the controls and the individuals with obesity. As shown in the volcano plot in Fig. 2, 48 of these proteins were significantly different, indicated in color and with protein names.

A weighted coexpression network analysis was conducted to cluster all the proteins from the Olink panel (Table S2) [17]. This was then used to determine correlations between the protein clusters and risk factors for metabolic syndrome and fat distribution and characteristics.

**Figure 4.**
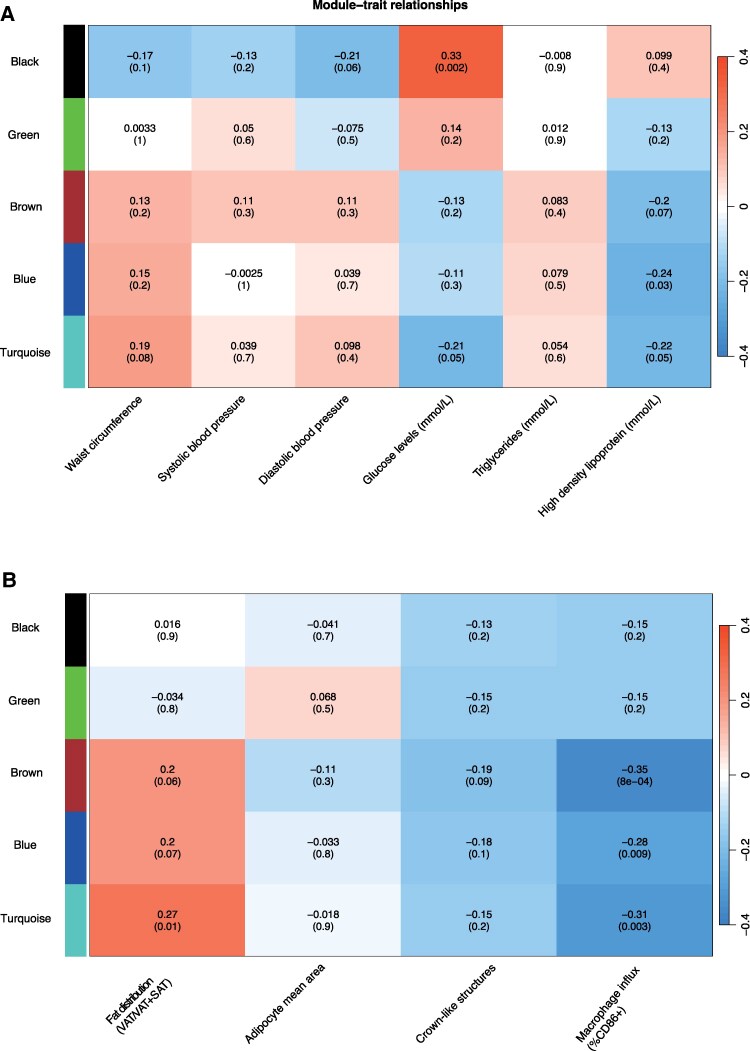
Clustering analysis of all the proteins shows that different protein clusters are associated with different risk factors in individuals with obesity. The correlation between the proteins and metabolic syndrome (A) or fat distribution (B) risk factors is shown as the number and the color within the boxes, while the *P*-value of the relationships is given between brackets. Rows signify the different protein clusters, and the columns are the common factors for metabolic syndrome.

To investigate the risk factors for obesity and significant proteins, the clustered proteins (Table S2) [17] were used in a correlation analysis (Fig. 4). Figure 4A shows the different metabolic syndrome risk factors and the correlation score of each module. An increase in the black cluster is associated with an increased concentration in blood glucose levels, while high-density lipoprotein is negatively associated with the blue and turquoise clusters. In addition, the clustered proteins were used in a correlation analysis with fat distribution and its characteristics (Fig. 4B). The turquoise cluster was associated with fat distribution (as measured and calculated using visceral adipose tissue/visceral adipose tissue + subcutaneous adipose tissue [VAT/VAT+SAT]). The influx of macrophages (%CD86+) were negatively associated with the turquoise, blue, and brown clusters.

**Figure 5.**
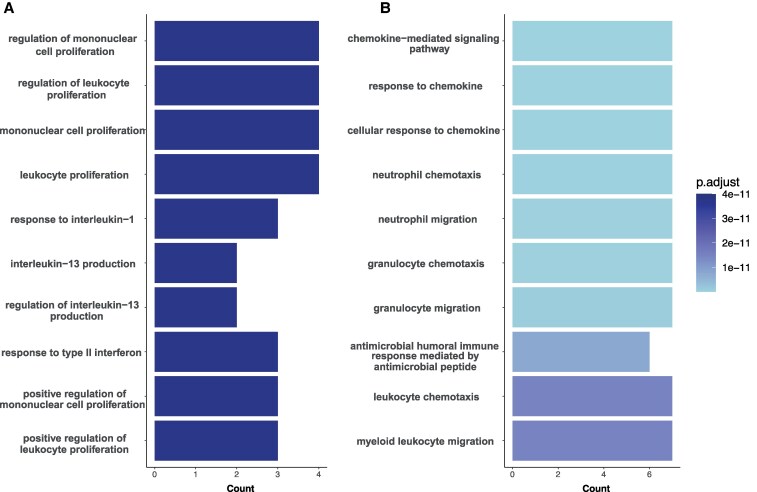
Overrepresentation analysis of the enriched proteins. Pathways that belong to the differentially expressed proteins are visualized for both the individuals without obesity (A) and the individuals with obesity (B). The counts represent the genes that are involved in the pathway. The *P*-value is adjusted using Benjamini-Hochberg post hoc test.

To understand the biological processes that are associated with the differentially expressed proteins, an ORA was used for a functional interpretation of the proteins of interest. The differentially expressed proteins from the volcano plot (Fig. 2) were used to create an ORA (Fig. 5). The results from the ORA show that different pathways are overrepresented between the normal-weight controls and the individuals with obesity (Fig. 5). The individuals with obesity show a much stronger overrepresentation (*P* < .001) compared to the control group, indicating that these pathways are more strongly associated.

## Discussion

The objective of this study was to explore the differences in inflammation proteins in the urine between normal-weight controls and individuals with obesity. Our findings show that several proteins are enriched in individuals with obesity. In addition, protein clustering analysis revealed different clusters that were associated with risk factors for metabolic syndrome and fat distribution. This indicates that a cluster of proteins can be a measure of different risk factors in obesity. Pathway analysis of a subset of the proteins revealed a strong overrepresentation of the pathways involved in chemokine-mediated pathways in individuals with obesity. These findings indicate that urinary measurements could provide a novel and noninvasive method of measuring inflammation in individuals with obesity.

Previous studies have shown that obesity can promote systemic inflammation in humans [25]. Urine can be used as a reflection of the serum proteome. Thus, proteins in the urine can be increased because of the systemic inflammation seen in obesity. However, it is possible that these proteins do not originate from the circulation but rather originate from the kidney, especially as obesity can affect kidney function through hypertension, which could lead to kidney damage and the release of cytokines and chemokines by resident immune cells in the kidney [26, 27]. Though this seems unlikely, previous research has shown that children who are overweight have an increase in urinary markers that are associated with inflammation, including IL-6 and CRP [28], indicating that the inflammatory markers can pass the glomerulus and enter the urine, which can be facilitated by kidney hyperfiltration in obese individuals [29]. Furthermore, other studies have shown the presence of inflammation markers in the urine during different cancers and during sepsis, further consolidating that inflammation in the body can be evaluated in the urine [30, 31]. The current study found associations between the proteins and risk factors of metabolic syndrome, which further support the idea that the urine could be used to determine the status of the circulation. According to this data, it is likely that the inflammatory markers can enter the urine. Facilitation of inflammatory markers in the urine could occur through endothelial dysfunction, causing increased vascular permeability and leakage into the urine [32]. Alternatively, extracellular vesicles originating from the adipose tissue can be a source of inflammatory markers, though this has not been proven in vivo yet.

The relationship between the clusters and the traits associated with metabolic syndrome is determined by investigating data relating to the traits, including waist circumference, blood pressure, glucose, and triglycerides, that were obtained from the 300OB cohort. The most important finding is the positive associations between some of the clusters and the metabolic syndrome risk factors. The brown clustered proteins are related to waist circumference, while the black cluster is associated with glucose levels. Furthermore, this study found that some of the clusters were correlated with characteristics of fat and fat distribution. The most interesting association was the negative association between the brown, blue, and turquoise cluster and %CD68, which is unexpected. The %CD68 represents the influx of macrophages into the fat tissue, and the blue cluster contains many CCL and CXCL proteins. These chemokines are often involved in cell chemotaxis, and they would be expected to be involved in obesity and low-grade inflammation. Thus, it was expected to have a positive correlation with the influx of macrophages [33]. A possible explanation for the negative association might be that these proteins that were found are not involved in the activation of macrophages or that they may not be as strong as potent inducers such as interferon-γ [34]. Furthermore, it could be that these proteins are released in very low amounts into the urine, creating an underestimation in the concentration of these cytokines and chemokines in the circulation. Another finding was that the turquoise cluster exhibited a strong association with fat distribution. As the turquoise clusters contain many different interleukins, this finding is consistent with previous research that has found associations between some interleukins in plasma and fat distribution, specifically with IL-1Ra and IL-6 [35, 36]. These findings indicate that the inflammatory proteins are at least partly a reflection of the circulation, as the proteins correlate with the different markers and traits from the circulation.

Further classification of the proteins revealed many pathways that are involved in individuals with obesity and in the healthy-weight controls. The main implicated pathway in the healthy-weight controls is the regulation of mononuclear cell proliferation. The negative effect of obesity on the function of mononuclear cells has been described before and leads to an increased sensitivity to infections [37, 38]. People with obesity also often exhibit leptin deficiency, which has been linked to reduced hematopoiesis and impaired immunity [39, 40]. Leptin, in addition to being a satiety hormone, also acts on bone marrow hematopoiesis [41], thus affecting the proliferation of different mononuclear cell types.

The current investigation found that individuals with obesity exhibit an increase in pathways related to chemokine-mediated processes. Chemokines are secreted molecules that mediate cell migration, and their expression is increased in adipose tissue of people with obesity [42, 43]. Chemokines promote the inflammatory environment in obesity by facilitating the influx of proinflammatory neutrophils and monocytes [44, 45]. The increased chemokines lead to the other top pathways found in our analysis, such as granulocyte chemotaxis. This has been shown to occur with high leptin concentrations [46]. In addition, increased leukocyte chemotaxis can also be attributed to high leptin concentrations. A previous study has shown that leukocyte count and plasma leptin concentration were indirectly and positively correlated [47]. Taken together, our findings show that the chemokine system is affected in obesity, which partly results in cell chemotaxis and the release of more inflammation by the infiltrated cells in the adipose tissue.

Our study has several strengths, including the comparability between the 2 cohorts in terms of age and sex. Limitations of the current study include the use of targeted proteins that are present in the Olink panel, which leads to a narrow view on the proteins that could be present in the urine. Furthermore, no data on the metabolic syndrome risk factors were present from the normal-weight controls, which could have provided a more complete view of the associations with serum markers. Lastly, little is known about how well the urine recapitulates the situation in the circulation, making it unclear whether the proteins that were found are present in the urine due to local effects or due to it being present in the circulation, though our results show that it is likely that the proteins at least partly originate from the circulation. Even though Olink only provides a targeted approach; it does allow a broad targeted search for the most common inflammatory markers, acting as foundation for this exploratory study.

This study set out to gain a better understanding of the urinary inflammatory proteome between individuals with obesity and healthy-weight individuals. This study has identified several significant and differentially expressed proteins and pathways that are implicated in obesity. Taken together, these findings suggest that multiple aspects of the immune function related to inflammation are affected. This is in accordance with other literature and suggests that urine could be used in determining inflammation in a noninvasive way. Ultimately, in the future, these biomarkers could be used to determine low-grade inflammation in individuals with obesity and identify those at high risk of developing comorbidities and complications. Further research is necessary to better identify and establish the found markers in an independent cohort and determine the sensitivity and specificity of these markers in distinguishing low-grade inflammation and metabolic syndrome. Furthermore, the added value of these urinary proteins should be investigated in addition to other traditional factors such as BMI. Little is known about the urinary proteome of people with obesity, and this study provides an overview of different proteins that are differentially expressed in these individuals. These insights could be used for further biomarker development or as a noninvasive measure of inflammation in obesity.

## Data Availability

Some or all datasets generated during and/or analyzed during the current study are not publicly available but are available from the corresponding author on reasonable request.

## Clinical Trial Information

300OB - NL46846.091.13 and 500FG - NL42561.091.12.
